# A PILOT STUDY OF EPIGENETIC AGING IN THE CONTEXT OF AN INTENSIVE LIFESTYLE INTERVENTION: THE LOOK AHEAD STUDY

**DOI:** 10.1093/geroni/igad104.1941

**Published:** 2023-12-21

**Authors:** Lindsay Reynolds, Mark Espeland, Timothy Howard, Carl Langefeld, Mara Vitolins, Lynne Wagenknecht

**Affiliations:** Wake Forest University School of Medicine, Winston-Salem, North Carolina, United States; Wake Forest University School of Medicine, Winston-Salem, North Carolina, United States; Wake Forest University School of Medicine, Winston-Salem, North Carolina, United States; Wake Forest University School of Medicine, Winston-Salem, North Carolina, United States; Wake Forest School of Medicine, Winston Salem, North Carolina, United States; Atrium Health/Wake Forest Baptist, Winston-Salem, North Carolina, United States

## Abstract

Type 2 diabetes and obesity increase the rate of accumulation of age-related health deficits and accelerate biological aging. An intervention promoting weight loss among adults with these conditions, the Action for Health in Diabetes (Look AHEAD) Intensive Lifestyle Intervention (ILI), attenuated health deficit accumulation measured by a frailty index, through a variety of diet and exercise strategies and optional weight loss medications to achieve and maintain ≥7% weight loss. To better understand the long-term effects of the Look AHEAD ILI on aging biology, we leveraged biospecimens from 62 women aged 60-71 years who participated in the Look AHEAD randomized clinical trial of ILI (n=32) and diabetes support and education (control condition, n=30), and tested for associations of ILI vs control condition on the rate of epigenetic aging. Five DNA methylation-based aging measures were generated from blood collected at baseline and 14 years later. Unexpectedly, the intervention was associated with a long-term increase in AgeAccelPheno compared to the control condition (t-test, p< 0.05). ILI was not associated with a change in other epigenetic measures over 14 years (AgeAccelHannum, AgeAccelHorvath, AgeAccelGrim and DunedinPACE), or a change in a frailty index reflecting accumulation of 38 health deficits. Overall, the average change in frailty index was higher among participants with advanced epigenetic age at baseline (AgeAccelHannum>0 and AgeAccelGrim>0,p < 0.05), suggesting that epigenetic aging markers may be useful predictors of frailty risk. Well-powered studies are needed to better understand the long-term impact of intensive lifestyle interventions on epigenetic aging in adults with diabetes and obesity.

